# The use of electronic medical records for recruitment in clinical trials: findings from the Lifestyle Intervention for Treatment of Diabetes trial

**DOI:** 10.1186/s13063-016-1631-7

**Published:** 2016-10-13

**Authors:** Valery S. Effoe, Jeffrey A. Katula, Julienne K Kirk, Carolyn F Pedley, Linda Y. Bollhalter, W. Mark Brown, Margaret R. Savoca, Stedman T. Jones, Janet Baek, Alain G. Bertoni, the LIFT Diabetes Research Team

**Affiliations:** 1 Department of Epidemiology and Prevention, Division of Public Health Sciences, Wake Forest School of Medicine, Medical Center Blvd, Winston-Salem, NC 27127 USA; 2 Department of Medicine, Morehouse School of Medicine, Atlanta, GA USA; 3 Department of Health and Exercise Science, Wake Forest University, Winston-Salem, NC USA; 4 Department of Family and Community Medicine, Wake Forest School of Medicine, Winston-Salem, NC USA; 5 Department of Internal Medicine, Wake Forest School of Medicine, Winston-Salem, NC USA; 6 Department of Biostatistical Sciences, Wake Forest School of Medicine, Winston-Salem, NC USA; 7 Department of Medicine, Wake Forest School of Medicine, Winston-Salem, NC USA; 8 Maya Angelou Center for Health Equity, Wake Forest School of Medicine, Winston-Salem, NC USA

**Keywords:** Type 2 diabetes, Recruitment methods, Electronic medical records, Lifestyle interventions, Community-based interventions, Randomized controlled trial, African Americans

## Abstract

**Background:**

The use of the electronic medical record (EMR) system in recruitment in clinical trials has the potential for providing a very reliable and cost-effective recruiting methodology which may improve participant recruitment in clinical trials. We examined a recruitment approach centered on the use of the EMR, as well as other traditional methods, in the Lifestyle Intervention for Treatment of Diabetes (LIFT Diabetes) trial.

**Methods:**

LIFT Diabetes is a randomized controlled trial designed to investigate the effects of two contrasting interventions on cardiovascular disease risk: a community-based intensive lifestyle program aimed at achieving weight loss and a clinic-based enhanced diabetes self-management program. Eligible participants were overweight/obese (body mass index, BMI ≥25 kg/m^2^) patients with type 2 diabetes who were aged 21 years or older. Recruitment strategies included the use of the EMR system (primary), direct referrals, media advertisements, and community screenings.

**Results:**

A total of 1102 telephone screens were conducted, resulting in randomization of 260 participants (61.5 % from EMR, mean age 56.3 years, 66.2 % women, 48.1 % non-Hispanic blacks) over a 21-month period, with a yield of 23.6 %. Recruitment yields differed by recruitment method, with referrals having the highest yield (27.5 %). A history of cardiovascular disease was the main health reason for exclusion from the study (16.5 %). An additional 8.9 % were excluded for BMI <25 kg/m^2^ (<27 kg/m^2^ for insulin users), 5.4 % could not exercise, 5.2 % had an HbA1c >11 %, and 34.9 % were excluded for other non-medical reasons. Exclusion criteria did not appear to differentially affect enrollment in terms of race or ethnicity.

**Conclusions:**

Future clinical studies should tailor their recruitment strategies based on the participant demographics of interest. Efficient methods such as using the EMR system and referrals should be prioritized over labor-intensive, low-yielding methods such as community screenings and mass mailings.

**Trial registration:**

ClinicalTrials.gov: NCT01806727. Registered on 5 March 2013.

**Electronic supplementary material:**

The online version of this article (doi:10.1186/s13063-016-1631-7) contains supplementary material, which is available to authorized users.

## Background

Recruitment of participants in clinical trials generally falls behind schedule in up to 86 % of all trials, with 13 % of trials behind by more than 6 months [1]. Early success in recruiting is a reliable predictor of the likelihood of completing a clinical trial [2]. The use of patient registries and databases to effectively recruit participants for clinical trials has increased in recent years [3–6]. The use of electronic medical records (EMRs) may improve participant recruitment by providing an efficient method for prescreening individuals based on predefined inclusion/exclusion criteria [7, 8]. In one community weight loss lifestyle trial which used almost exclusively medical record review to recruit overweight adults with diabetes, the recruitment yield was 21.5 % [4].

Barriers to the enrollment of participants in clinical trials include factors related to healthcare providers, such as a lack of interest in and knowledge about the trial, physician bias about the therapy under investigation, and concerns about losing the patient to follow-up [9]. Factors related to participants can also affect enrollment [10]. For example, exclusion criteria that are too stringent may differentially affect enrollment of particular race/ethnic groups.

The purpose of this report is to (1) compare the recruitment yield from the use of the EMR system versus traditional recruitment methods for the LIFT Diabetes trial, (2) evaluate the direct cost of recruitment, and (3) examine the differential effects of screening exclusion criteria on the enrollment of participants from the different race/ethnic groups.

## Methods

### Study design

The LIFT Diabetes randomized trial was designed to investigate the effects of two contrasting interventions (a 12-month community-based intensive lifestyle intervention versus a clinic-based enhanced diabetes self-management program) on cardiovascular disease (CVD) risk in overweight and obese adults with type 2 diabetes [11, 12].

The trial protocol was approved by the Institutional Review Board (IRB) of the Wake Forest School of Medicine, and a limited temporary waiver of the Health Insurance Portability and Accountability Act (HIPAA) [13] authorization was issued by the IRB, which permitted study investigators to access protected health information to confirm eligibility and facilitate initial contact for recruitment as allowed by government regulations. All participants in the study gave a verbal consent before the telephone screen and a written informed consent at their initial screening visit.

### Study eligibility

Participants eligible for recruitment into the LIFT Diabetes trial were aged 21 or older, had a confirmed diagnosis of type 2 diabetes (HbA1c ≥6.5 %, a physician diagnosis of diabetes on the participant’s problem list, or the use of diabetes medication), a body mass index (BMI) of 25 or greater (27 or greater if on insulin), and a regular source of medical care. Participants were excluded because of the following: history of prior cardiovascular disease or history of cancer with expected survival less than 2 years; glycosylated hemoglobin (HbA1c) >11 %, blood pressure >160/100 mmHg, triglycerides >600 mg/dl; history of prior weight loss surgery; unstable psychiatric disease; inability to walk two blocks without stopping; drug or alcohol abuse; use of drugs known to affect body weight (e.g., corticosteroids); pregnancy or breastfeeding; and advanced renal disease (estimated glomerular filtration rate, eGFR <45 ml/min/1.73 m^2^).

### Recruitment methods

The LIFT Diabetes trial aimed to recruit a sample of 55–60 % African American, 10–15 % Hispanic, and 25–35 % non-Hispanic White (NHW) participants. The trial recruited 260 participants in Winston-Salem, North Carolina, USA and surrounding areas.

The primary recruitment method employed was the use of the EMR system and targeted mailing. Individuals with a diagnosis of diabetes mellitus on the problem list in their medical records were identified via an EMR system containing records for Wake Forest Baptist Health (WFBH) patients. These individuals were then initially screened electronically (e-screening) for major exclusions (history of CVD, cancer, prior weight surgery) cited on the problem list. During this initial e-screening phase, a less strict cut-off value was used for clinical and biological parameters such as BMI, blood pressure, HbA1c, triglycerides, and eGFR, since these measurements can change significantly over time. For example, for blood pressure, a systolic pressure of 170 mmHg and/or a diastolic pressure of 110 was used for e-screening, in place of the predefined cut-off of >160/100 mmHg. Individuals remaining potentially eligible were mailed a brochure with an opt-out postcard to return if not interested. If a postcard was not received after 2 weeks, a study staff member reached out to potential participants to determine interest and conduct a telephone screen. To assess the efficacy of the EMR as a screening tool, sensitivity and specificity analyses were done on a random subsample of participants with diabetes on the problem list.

Another recruitment method used was direct referrals from physicians or other healthcare providers, study participants, and study team members. To foster referrals from within the WFBH system, several study investigators made presentations at Internal Medicine and Family Medicine faculty meetings. For healthcare provider referrals, LIFT Diabetes contact cards were made available for interested individuals to provide their contact information.

Study team members attended six health screening events in the community (health fairs and church screenings), where interested participants were either screened on site or contacted at a later date by telephone. The study team also distributed flyers in targeted local pharmacies in the community and different WFBH medical practices. In order to increase the representation of African Americans in the trial, especially African American men, television advertisements were aired during specific programs and specific times of the day, and a study investigator completed a radio interview. LIFT Diabetes study advertisements were also printed and published in a local newspaper and magazine and in church bulletins, and posted online (on the Wake Forest Baptist Health website for clinical studies and an online press). All study recruitment materials were approved by the IRB of the Wake Forest School of Medicine and focused on three main criteria: adults aged 21 and older, a diagnosis of type 2 diabetes, and being overweight or obese.

### Participant screening and randomization

Participants who expressed interest in the study were screened either via telephone or face to face using a scripted screening instrument. Five telephone screening calls were attempted for each participant who did not opt out, and a voicemail message requesting a return call was recorded whenever possible. To determine potential eligibility during the phone screen, major exclusions were assessed. A typical telephone screen lasted on average 15 min. For most participants, eligibility to progress to the next screening phase was established during the telephone interview. For a few, a second review by the study physician was required.

All participants deemed potentially eligible after the telephone screen were invited to attend a clinic where additional screening was performed to assess final eligibility. During the clinic visit, the following eligibility parameters were assessed: confirmed diagnosis of diabetes, blood pressure ≤160/100 mmHg, BMI ≥25 kg/m^2^, urine (dipstick analysis) proteinuria <4+, HbA1c ≤11 %, eGFR ≥45 ml/min, triglycerides ≤600 mg/dl, absence of a history of CVD, ability to exercise (using the Physical Activity Readiness Questionnaire), and absence of severe depression (using the Patient Health Questionnaire - 9 instrument). Participants who remained eligible after the first clinic screening visit were invited to a baseline visit, during which they were randomized to one of the two arms of the trial. The duration of recruitment and randomization was dependent on the number of personnel, the duration of clinic visits, and the willingness of eligible individuals to participate. Of note, we aimed to recruit participants in waves, as we were limited to a maximum of six concurrent groups due to staffing and space constraints.

### Estimation of recruitment time

Personnel effort was estimated using full-time equivalents (FTEs) for all persons involved with recruitment. An FTE was estimated based on the percentage effort allocated to the activity of recruitment. For example, an FTE of 1.0 was equivalent to a 100 % effort, which translated to 40 h of work per week by the personnel. The number of persons directly involved with recruitment was not constant throughout the entire period, and as such personnel effort was estimated on a monthly basis. The actual amount of time (in hours) spent by personnel during recruitment was estimated. Estimates were done for the telephone screening encounter as well as both clinic visits. For the telephone screening encounters, the estimated time included the time to obtain a verbal consent, complete the screening questionnaire, and enter the data into the online database system. For both clinic visits (eligibility screen and baseline), the estimated time included the time to obtain a written consent, complete all relevant forms, and enter the data into the online database system.

### Statistical analyses

Results are presented as mean and standard deviation or number (percentage) where appropriate. Differences in demographic and clinical variables between enrolled and non-enrolled participants were assessed using the Kruskal-Wallis test for continuous variables and the chi-square or Fisher’s exact test (where cell counts are low) for categorical variables. The estimate for the overall recruitment yield was calculated as the ratio of participants enrolled in the study to the total number of participants reached by telephone. The recruitment yield was also estimated per recruitment method and by race/ethnic group. (The cumulative number of participants randomized against the study goal is plotted in Fig. 2.) To test if exclusion criteria differentially affected enrollment of African Americans, logistic regression models were fitted to estimate odds ratios for African Americans compared to other race/ethnic groups (as the reference) for each of the major criteria. For this analysis, 41 participants were excluded because they had missing data on race. A *P* value of less than 0.05 was considered statistically significant. All analyses were performed using SAS version 9.3 (SAS Institute Inc., Cary, NC).

## Results

### Recruitment stages

**Fig. 1:**
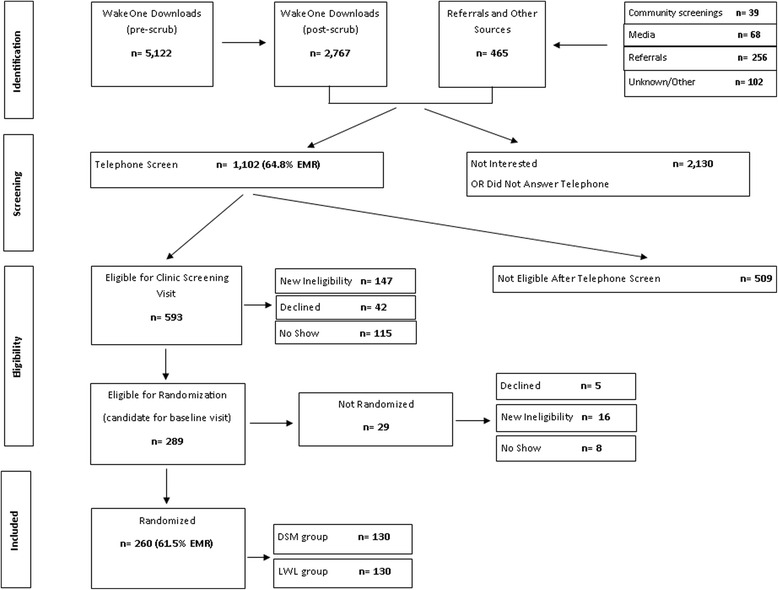
Flowchart of the recruitment process for the LIFT Diabetes trial. *Community screenings* refers to screenings during health and church fairs. *Media* refers to television, radio, and print advertisements. *Referrals* were from healthcare providers, study staff, participants’ friends and relatives, and other studies. *Unknown/other* refers to participants whose source of recruitment was either unknown or from the Wake Forest Be Involved website, ClinicalTrials.gov website, and other online advertisement. *DSM* diabetes self-management, *LWL* lifestyle weight loss

**Fig. 2:**
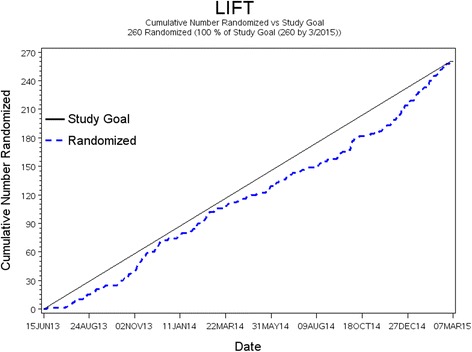
Cumulative number of participants randomized versus the study goal in the LIFT Diabetes trial (period from June 2013 to March 2015). The *solid line* represents the study goal, while the *dashed line* is a plot of the actual number randomized for the study

Recruitment efforts began in May 2013 and were concluded by the first week in March 2015. A total of 260 participants were randomized over a 21-month period (June 2013 to March 2015). The recruitment process was completed in four phases. In the first phase, a total of 5122 unique medical records (after duplicate records had been eliminated) were abstracted from the EMR database and assessed for major exclusions. After this initial assessment, 2767 EMR records were retained for a telephone screen. An additional 465 telephone screen-eligible participants came from direct referrals, media advertisements, community screening events, and other sources (Fig. 1). In the second phase, 1102 (64.8 % from EMR) participants contacted completed a telephone screen (a total of 6899 phone calls were made, including missed calls), of which 593 (53.8 %) qualified for a clinic screening visit (third phase of recruitment). Of the 593 potential participants, 289 were declared eligible for a baseline visit and eventual randomization. In the last phase of recruitment (randomization phase), 260 (61.5 % from EMR) participants were randomized (130 in each arm of the study). Figure 2 displays the cumulative number of participants randomized versus the study goal.

### Characteristics of excluded participants

Among all individuals reached by telephone for an initial screen, 842 (76.4 %) were excluded from the study. At the level of the telephone screen, compared to enrolled participants, a higher percentage of non-Hispanic blacks (NHB) were excluded (57.3 versus 48.1 %, *P* = 0.01) (see Table 1). More non-Hispanic whites (NHW) were enrolled than excluded at this stage of screening (45.4 versus 34.1 %). At the level of the clinic screening visit, compared to those who enrolled, excluded individuals were significantly younger, and a lower percentage were on medications for diabetes, hypertension, and dyslipidemia (Table 1). There were no differences in sex, BMI, HbA1c, duration of diabetes, blood pressure, or smoking status between enrolled participants and those excluded.

**Table 1:** Demographic and clinical characteristics of LIFT Diabetes participants by enrollment status

Characteristic	Total screened participants	Non-enrolled participants	Enrolled participants	*P* value
Phone screen eligible, *N*	1102	842	260	
Age, years	55.0 ± 11.6	54.7 ± 11.8	56.3 ± 10.7	0.054
Female, *n* (%)	674 (62.9)	502 (61.8)	172 (66.2)	0.21
Race/ethnicity				0.012
Non-Hispanic black	584 (55.0)	459 (57.3)	125 (48.1)	
Non-Hispanic white	391 (36.9)	273 (34.1)	118 (45.4)	
Hispanic	31 (2.9)	25 (3.1)	6 (2.3)	
Other (NA, AN, AI, Asian, PI)	55 (5.2)	44 (5.5)	11 (4.2)	
Screening visit eligible, *N*	407	147	260	
Age, years	55.3 ± 10.7	53.7 ± 10.3	56.3 ± 10.7	0.033
Female, *n* (%)	264 (64.9)	92 (62.6)	172 (66.2)	0.47
Race/ethnicity				0.054
Non-Hispanic black	206 (50.6)	81 (55.1)	125 (48.1)	
Non-Hispanic white	168 (41.3)	50 (34.0)	118 (45.4)	
Hispanic	15 (3.7)	9 (6.1)	6 (2.3)	
Other (NA, AN, AI, Asian, PI)	18 (4.4)	7 (4.8)	11 (4.2)	
Smoking status, *n* (%)				0.44
Current	67 (16.8)	27 (19.4)	40 (15.4)	
Former	125 (31.3)	39 (28.1)	86 (33.1)	
Never	207 (51.9)	73 (52.5)	134 (51.5)	
Body mass index, kg/m^2^	38.1 ± 8.8	38.7 ± 9.3	37.8 ± 8.5	0.33
Body mass index categories, kg/m^2^				0.65
25–29.9	74 (18.3)	24 (16.7)	50 (19.2)	
30–34.9	125 (30.9)	41 (28.5)	84 (32.3)	
35–39.9	88 (21.8)	35 (24.3)	53 (20.4)	
≥ 40	117 (29.0)	44 (30.6)	73 (28.1)	
Diabetes medication use, *n* (%)	325 (79.9)	100 (68.0)	225 (86.5)	<0.0001
Hypertension medication use, *n* (%)	303 (74.5)	94 (63.9)	209 (80.4)	<0.001
Lipid-lowering medication use, *n* (%)	208 (51.1)	60 (40.8)	148 (56.9)	0.002
HbA1c	7.8 ± 1.7	8.1 ± 2.1	7.6 ± 1.3	0.57
Duration of diabetes, years	8.6 ± 8.0	8.8 ± 8.5	8.5 ± 7.8	0.99
Estimated GFR, ml/min/1.73 m^2^	92.4 ± 22.2	93 ± 22	92.1 ± 22.3	0.23
Systolic blood pressure, mmHg	127.1 ± 16.7	128.6 ± 18.4	126.3 ± 15.7	0.37
Diastolic blood pressure, mmHg	77.0 ± 10.4	78.8 ± 10.3	76.1 ± 10.3	0.046
Triglycerides, mg/dl	169.8 ± 106.0	172.3 ± 114.2	168.5 ± 101.7	0.88

Data are mean ± SD or number (percentage)

*AI* American Indian, *AN* Alaskan native, *GFR* glomerular filtration rate, *NA* Native American, *PI* Pacific Islander

Table 2 illustrates the reasons for exclusion of individuals during the LIFT Diabetes screening process. The major health reason for exclusion was a history of CVD or cardiovascular procedure (16.5 % of participants). A BMI <25 kg/m^2^ (<27 kg/m^2^ for insulin users) and an HbA1c > 11 % accounted for more than 8.9 and 5.2 % of exclusions, respectively. About 5.4 % of those screened reported being unable to exercise. In multivariable logistic regression analysis, compared to other race/ethnic groups, NHB were not more likely to be excluded from the study because of predefined criteria (Additional file 1: Table S1).

**Table 2:** Exclusion criteria by race/ethnic group at the time of the prescreen and clinic screening visit in the LIFT Diabetes trial

	Total screened (*N* = 801)	African Americans (*N* = 459)	Other race/ethnic groups (*N* = 342)	*P *value
Non-diabetic	15 (1.9 %)	5 (1.1 %)	10 (2.9 %)	0.07
Under 25 and always on insulin	7 (0.9 %)	7 (1.5 %)	0 (0 %)	0.02
BMI <25 kg/m^2^ or <27 kg/m^2^ for insulin users	71 (8.9 %)	42 (9.2 %)	29 (8.5 %)	0.80
History of CVD or cardiovascular procedure*	132 (16.5 %)	70 (15.3 %)	62 (18.1 %)	0.29
Alcohol (>14 drinks for men aged 65 years or less, > 7 drinks for men and women aged over 65 years) or drug abuse	11 (1.4 %)	2 (0.4 %)	9 (2.6 %)	0.01
Other medical conditions (including chronic disease, leg amputation, blood clot, IBD, Crohn’s disease, acromegaly)	43 (5.4 %)	31 (6.8 %)	12 (3.5 %)	0.06
Prior weight loss surgery	19 (2.4 %)	12 (2.6 %)	7 (2 %)	0.65
History of cancer	4 (0.5 %)	2 (0.4 %)	2 (0.6 %)	1.00
Unable to exercise	43 (5.4 %)	29 (6.3 %)	14 (4.1 %)	0.21
Use of steroid pills or shots	33 (4.1 %)	18 (3.9 %)	15 (4.4 %)	0.86
Unwilling to stop weight loss medications/program	13 (1.6 %)	6 (1.3 %)	7 (2 %)	0.42
Pregnancy, breastfeeding	6 (0.7 %)	5 (1.1 %)	1 (0.3 %)	0.25
Hospitalized depression/PHQ-9	11 (1.4 %)	3 (0.7 %)	8 (2.3 %)	0.06
Physician review includes PAR-Q	53 (6.6 %)	31 (6.8 %)	22 (6.4 %)	0.89
Blood pressure ≥160/100 mmHg	8 (1.0 %)	5 (1.1 %)	3 (0.9 %)	1.00
HbA1c ≥11 %	42 (5.2 %)	25 (5.4 %)	17 (5 %)	0.87
Laboratory exclusions (GFR <45, triglycerides >600)	10 (1.2 %)	3 (0.7 %)	7 (2 %)	0.11
Other reasons (household member works for LIFT, cannot commit to travel, another research study, schedule conflict, no PCP, needs sign language interpreter, ineligible with no reason given)	109 (13.6 %)	59 (12.9 %)	50 (14.6 %)	0.47
Decided not to participate/no show	171 (21.3 %)	104 (22.7 %)	67 (19.6 %)	0.34

*Includes: MI, stroke, TIA, coronary bypass surgery, coronary angioplasty, heart balloon surgery, stenting, and cardiac rehabilitation

*BMI* body mass index, *CVD* cardiovascular disease, *GFR* glomerular filtration rate, *HbA1c* glycosylated hemoglobin, *IBD* inflammatory bowel disease, *MI* myocardial infarction, *PAR-Q* physical activity readiness questionnaire, *PCP* primary care physician, *PHQ-9* patient health questionnaire, *TIA* transient ischemic attack

### Recruitment yield by race/ethnicity and by recruitment method

**Fig. 3:**
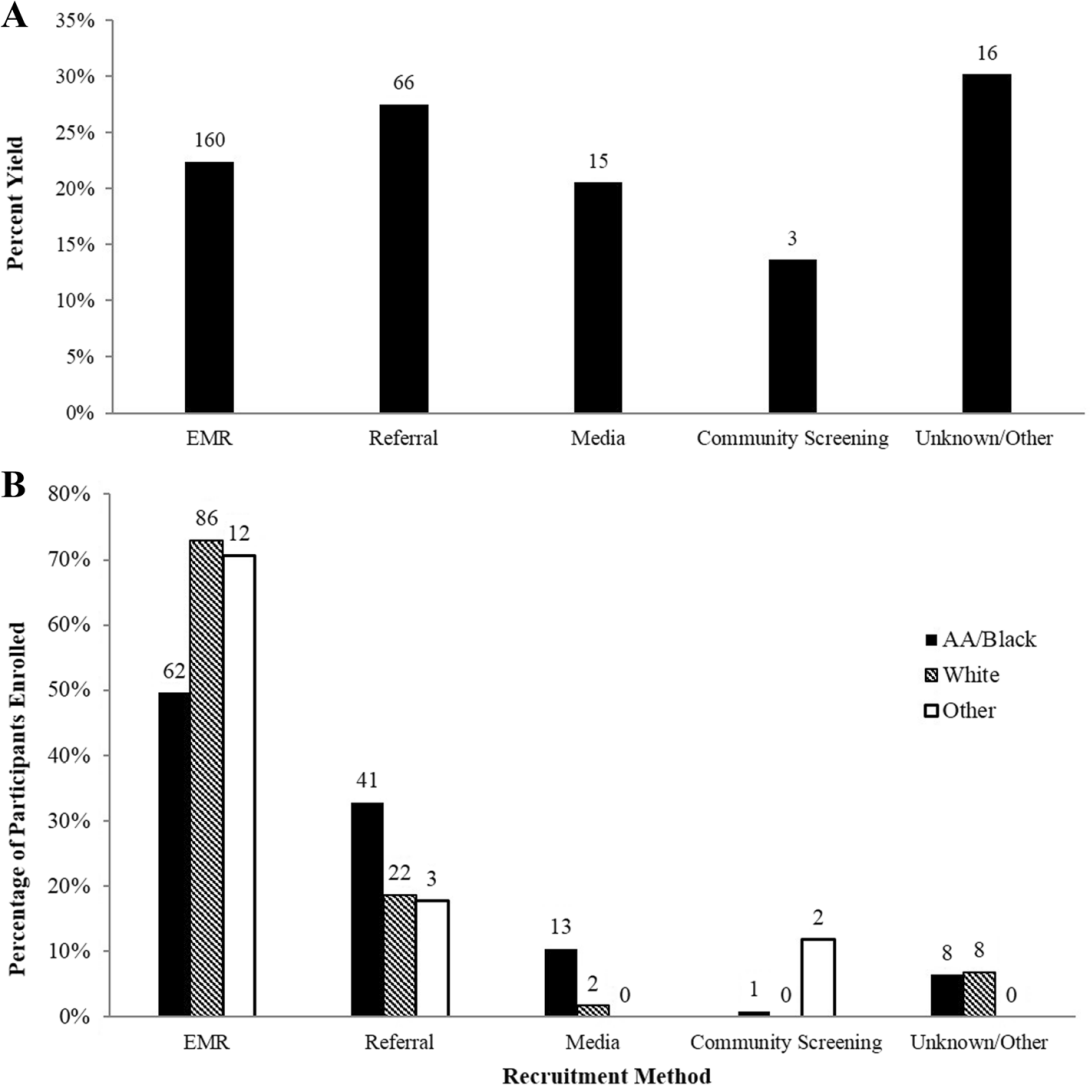
**a** Recruitment percent yield. **b** Percentage of participants enrolled. Data are presented by recruitment method. The *horizontal axis* displays the different recruitment methods. The *numbers* on the bars indicate the actual number of participants enrolled for each method. *Community screening* refers to screenings during health and church fairs. *Media* refers to television, radio, and print advertisements. *Referrals* were from healthcare providers, study staff, participants’ friends and relatives, and other studies. *Unknown/other* refers to participants whose source of recruitment was either unknown or from the Wake Forest Be Involved website, ClinicalTrials.gov website, and other online advertisement. *AA* African American, *EMR* electronic medical record

Overall, the recruitment yield was 23.6 %. The yield was higher for NHW (30.2 %), compared to NHB (21.4 %) and Hispanics (20 %). When recruitment methods were compared, the yield was highest for referrals (27.5 %). The yield for participants recruited through the EMR was 22.4 %, higher than that for community screening events (13.6 %) and media advertisements (20.5 %) (Fig. 3a).

### Randomized participants by race/ethnicity and by recruitment method

Overall, 61.5 % of randomized participants were recruited through the EMR system, compared to 25.4 % recruited through direct referrals. About two-thirds of all participants recruited via referrals were NHB. A smaller percentage of randomized participants were recruited through media advertisements (5.8 %), health and church screening events (1.2 %), and other methods (6.1 %). Figure 3b displays the percent of randomized participants by recruitment method and by race/ethnicity. There was a significance difference in proportions of NHW, NHB, and other race/ethnic groups by the recruitment methods (*P* < 0.0001). About three-quarters (72.9 %) of randomized NHW were recruited through the EMR, compared to 49.6 % of randomized NHB (Fig. 3b). In comparison, 32.8 and 10.4 % of randomized NHB, versus 18.6 and 1.7 % of randomized NHW, were recruited through direct referrals and media advertisements, respectively.

### Estimation of recruitment time

Average monthly personnel effort throughout the recruitment period ranged from 0.613 to 0.825 FTE per week (24.5–33.0 h per week) per personnel. The average time spent to complete a telephone screen was 0.5 h (30 min). A total of 1102 telephone screens were completed, which amounts to an average of 551 h. Clinic visits lasted on average 2 h each. There were a total of 478 completed eligibility screening visits and 281 completed baseline visits. The total time spent for both clinic visits averaged 1518 h. Per recruited participant, the average time spent was 4.5 h.

For the 2130 uncompleted telephone screens (either due to a nonresponse to the telephone call or because the participant expressed no interest in participating), each encounter attempt lasted on average 5 min or less. These time estimates do not take into account the time spent for unanswered telephone call attempts, telephone calls to schedule clinic appointments, and time spent designing brochures/flyers and media advertisements.

### Sensitivity analysis

On a random subsample of participants (*n* = 200, 27 % African Americans), the sensitivity and specificity of the EMR as a screening tool were assessed. Eligibility of participants based on the EMR was checked against eligibility based on a manual review of patients’ medical charts. The sensitivity of the EMR was estimated at 45.3 % and its specificity at 91.2 %.

## Discussion

The goal of recruitment for the LIFT Diabetes trial was to enroll 260 participants, including a target for minority groups higher than that of previous similar trials (55–60 % NHB and 10–15 % Hispanics). Using the EMR as the primary method of recruitment, the recruitment goal was achieved within close to the prespecified time period (recruitment was extended by one week). Our analysis also revealed a recruitment yield of more than 24 % and a total direct cost of $821 to randomize each enrolled participant. The main medical reasons for exclusion were a history of CVD or cardiovascular procedure and a BMI <25 kg/m^2^.

Our recruitment period of 21 months (approximately 12 participants randomized per month) is consistent with recruitment in other lifestyle intervention studies. In Healthy Living Partnerships to Prevent Diabetes (HELP PD) and the multisite Lifestyle Interventions and Independence for Elders (LIFE) Study, over a 21-month period, 301 participants (14/month) and 1635 elderly participants (10/month) were enrolled, respectively [14, 15]. Similarly, the multisite Look AHEAD (Action for Health in Diabetes) trial enrolled 5145 participants (10/month) over a period of 33 months [16, 17]. Although our recruitment rate was consistent with these studies, comparisons across studies can be challenging due to differences in recruitment goals and resources.

Overall, 23.6 % of all participants screened were enrolled into the study. The recruitment yield was higher for NHW (30.2 %) than for NHB (21.4 %) and Hispanics (20 %). Prior studies have reported lower recruitment yields ranging from 2.4–17.9 % [14, 15, 17–19]. In all of these studies, only traditional recruitment methods were used, including direct mass mailings. In LIFT Diabetes, the use of the EMR and e-screening permitted targeted mailing to only potentially eligible individuals for a telephone screen, thus increasing the overall yield. Similar to our finding, Parra-Medina D et al. recruited 189 participants into a weight management trial almost exclusively by medical record review and achieved a recruitment yield of 21.5 % [4]. We also observed a higher recruitment yield for African Americans in LIFT Diabetes compared to what was reported in Look AHEAD (21.4 % versus 12.9 %) [17].

The recruitment yield for the EMR was 22.4 %, consistent with the literature, which shows that recruitment using databases and patient registries tends to be more efficient than other methods [20]. More than two-thirds (61.5 %) of enrolled participants were drawn from the EMR, higher than that reported by direct mass mailings in the Diabetes Prevention Program (DPP) (29 %) and LIFE Study (57.9 %) [15]. The higher percentage observed with the EMR may be due to the initial e-screen, which yielded a narrow pool of potentially eligible participants for targeted mailings. Average monthly personnel effort throughout the recruitment period ranged from 0.613 to 0.825 FTE per week (24.5–33.0 h per week) per personnel. The total time spent to recruit and randomize each participant was on average 4.5 h.

The most challenging aspect of recruitment was enrolling a representative sample of African Americans, especially men. We found referrals and media advertisements to be effective strategies in addressing this challenge. This is consistent with prior research which shows that recruitment strategies using previous relationships and interpersonal approaches such as provider or healthcare professional referrals are generally efficient and effective in recruiting minorities [21, 22]. Our sample comprised 48 % NHB, 33 % of whom were drawn from direct referrals. In an effort to increase the representation of NHB, targeted radio and TV advertisements were aired during the last quarter of the recruitment window. Of the 15 participants recruited via media advertisements, 13 (86.7 %) were NHB. Participants had to be fluent in the English language, since all study materials and the interventions were conducted exclusively in English. As such, a majority of Hispanics were excluded from the study.

Recruitment via community health screening events had the lowest yield of all methods. In addition, these community screenings were very labor-intensive and expensive; only three participants were enrolled via this method, yet the cost to randomize one participant was more than double that of the EMR. Community screenings have been identified as the least successful of recruitment strategies in other similar clinical studies [18], suggesting that these should not be used as a primary strategy.

Despite the efficiency and cost-effectiveness of the EMR method, there are a number of limitations to using this approach. First, because the EMR was our primary recruitment method, we may have targeted mainly patients with type 2 diabetes who use the WFBH clinical centers as their source of care. The potential for this is however reduced, as slightly more than a third of our final sample came from methods other than the EMR. Also, WFBH has several clinics in Forsyth County and serves a significant proportion of the population. Second, the success of the EMR method is dependent on a proper medical coding system. Improper diagnosis coding may lead to misclassification, which can reduce the efficiency of the process. The EMR system used at the WFBH clinical centers allows the tagging of specific diagnosis codes, which makes e-screening feasible. Third, because the EMR system is not standardized across hospitals and clinical research sites, comparisons of EMR use in recruitment across studies can be challenging. Finally, the use of EMR for recruiting may miss potential participants who are underserved and have limited access to the healthcare system.

## Conclusions

Findings from the recruitment process in the LIFT Diabetes trial demonstrate that the EMR is an efficient tool which should be considered as a primary recruitment method for future clinical trials. Our results also show that the EMR approach should be used in combination with other recruitment strategies with proven efficacy in minority groups, such as direct referrals and media advertisements, as this can remarkably increase the recruitment yield for these groups. Future clinical studies will need to tailor their recruitment strategies based on the participant demographics of interest. Efficient methods with modest yields such as the EMR, direct referrals, and media advertisements should be prioritized over labor-intensive, low-yielding methods such as community screenings.

## Additional file

Additional file 1: Table S1. Differential effects of exclusion criteria on the enrollment of African Americans. (DOCX 19 kb)

## Abbreviations

AI: American Indian
BMI: Body mass index
CVD: Cardiovascular disease
DPP: Diabetes Prevention Program
eGFR: Estimated glomerular filtration rate
EMR: Electronic medical record
HbA1c: Glycosylated hemoglobin
HELP PD: Healthy Living Partnerships to Prevent Diabetes
LIFE Study: Lifestyle Interventions and Independence for Elders Study
Look AHEAD: Action for Health in Diabetes
NA: Native American
NHB: Non-Hispanic black
NHW: Non-Hispanic white
PI: Pacific Islander
WFBH: Wake Forest Baptist Health
