# Supplementary material for: The use of electronic medical records for recruitment in clinical trials: findings from the Lifestyle Intervention for Treatment of Diabetes trial
Source: Trials. 2016 Oct 13;17:496. doi: 10.1186/s13063-016-1631-7 (PMC5062894; doi:10.1186/s13063-016-1631-7)
Supplement: Additional file 1: Table S1. — Differential effects of exclusion criteria on the enrollment of African Americans. (DOCX 19 kb) [file 13063_2016_1631_MOESM1_ESM.docx]

Table S1: Differential effects of exclusion criteria on the enrollment of African Americans

|  | **Race/Ethnicity** | **Participants excluded, %** | **OR** | **95% CI** | **P-interaction** |
| --- | --- | --- | --- | --- | --- |
| Men | AA | 73 (51.4%) | 0.76 | (0.5, 1.2) | 0.23 |
|  | Non-AA | 74 (44.6%) |  |  |  |
| Women | AA | 127 (40.1%) | 1.01 | (0.7, 1.5) | 0.95 |
|  | Non-AA | 71 (40.3%) |  |  |  |
| Non Diabetic | AA | 5 (1.1%) | 2.73 | (0.8, 10.3) | 0.07 |
|  | Non-AA | 10 (2.9%) |  |  |  |
| BMI < 25 kg/m^2^ or < 27 kg/m^2^ for insulin users | AA | 7 (1.5%) | 0.00 | (0, 0.7) | 0.02 |
|  | Non-AA | 0 (0%) |  |  |  |
| Under 25 and always on Insulin | AA | 42 (9.2%) | 0.92 | (0.5, 1.6) | 0.80 |
|  | Non-AA | 29 (8.5%) |  |  |  |
| History of CVD or cardiovascular procedure | AA | 70 (15.3%) | 1.23 | (0.8, 1.8) | 0.29 |
|  | Non-AA | 62 (18.1%) |  |  |  |
| Alcohol or drug abuse | AA | 2 (0.4%) | 6.18 | (1.3, 59) | 0.01 |
|  | Non-AA | 9 (2.6%) |  |  |  |
| Other medical conditions (includes chronic disease, leg amputation, blood clot, IBD, Crohn’s disease, acromegaly) | AA | 31 (6.8%) | 0.50 | (0.2, 1) | 0.06 |
|  | Non-AA | 12 (3.5%) |  |  |  |
| Prior weight loss surgery | AA | 12 (2.6%) | 0.78 | (0.3, 2.2) | 0.65 |
|  | Non-AA | 7 (2%) |  |  |  |
| History of Cancer | AA | 2 (0.4%) | 1.34 | (0.1, 18.6) | 1.00 |
|  | Non-AA | 2 (0.6%) |  |  |  |
| Unable to exercise | AA | 29 (6.3%) | 0.63 | (0.3, 1.3) | 0.21 |
|  | Non-AA | 14 (4.1%) |  |  |  |
| Use of steroid pills or shots | AA | 18 (3.9%) | 1.12 | (0.5, 2.4) | 0.86 |
|  | Non-AA | 15 (4.4%) |  |  |  |
| Unwilling to stop weight loss medications/program | AA | 6 (1.3%) | 1.58 | (0.4, 5.7) | 0.42 |
|  | Non-AA | 7 (2%) |  |  |  |
| Pregnancy, breastfeeding | AA | 5 (1.1%) | 0.27 | (0, 2.4) | 0.25 |
|  | Non-AA | 1 (0.3%) |  |  |  |
| Hospitalized Depression/PHQ9 | AA | 3 (0.7%) | 3.64 | (0.9, 21.4) | 0.06 |
|  | Non-AA | 8 (2.3%) |  |  |  |
| Physician Review includes PARQ | AA | 31 (6.8%) | 0.95 | (0.5, 1.7) | 0.89 |
|  | Non-AA | 22 (6.4%) |  |  |  |
| Blood pressure ≥ 160/100 mmHg | AA | 5 (1.1%) | 0.80 | (0.1, 4.2) | 1.00 |
|  | Non-AA | 3 (0.9%) |  |  |  |
| HbA1c ≥ 11% | AA | 25 (5.4%) | 0.91 | (0.5, 1.8) | 0.87 |
|  | Non-AA | 17 (5%) |  |  |  |
| Laboratory Exclusions (GFR < 45, triglycerides > 600) | AA | 3 (0.7%) | 3.18 | (0.7, 19.1) | 0.11 |
|  | Non-AA | 7 (2%) |  |  |  |
| Other reasons (household member works for LIFT, Cannot commit to travel, another research study, schedule conflict, No PCP, needs sign language interpreter, ineligible with no reason given) | AA | 59 (12.9%) | 1.16 | (0.8, 1.8) | 0.47 |
|  | Non-AA | 50 (14.6%) |  |  |  |
| Decided Not to Participate/No Show | AA | 104 (22.7%) | 0.83 | (0.6, 1.2) | 0.34 |
|  | Non-AA | 67 (19.6%) |  |  |  |

AA indicates African American; BMI, body mass index; CI, confidence interval; CVD, cardiovascular disease; HbA1c, glycosylated hemoglobin; MI, myocardial infarction; OR, odds ratio; TIA, transient ischemic attack.
